# Freeze-Drying of Encapsulated Bacteriophage T4 to Obtain Shelf-Stable Dry Preparations for Oral Application

**DOI:** 10.3390/pharmaceutics15122792

**Published:** 2023-12-17

**Authors:** Paulina Śliwka, Grzegorz Skaradziński, Izabela Dusza, Aleksandra Grzywacz, Aneta Skaradzińska

**Affiliations:** Department of Biotechnology and Food Microbiology, Faculty of Biotechnology and Food Science, Wrocław University of Environmental and Life Sciences, 50-375 Wrocław, Polandgrzegorz.skaradzinski@upwr.edu.pl (G.S.);

**Keywords:** phage therapy, phage stability, phage formulation, immobilization of bacteriophages, bacteriophage lyophilization, bacteriophage encapsulation, mannitol

## Abstract

Therapeutic application of bacterial viruses (phage therapy) has in recent years been rediscovered by many scientists, as a method which may potentially replace conventional antibacterial strategies. However, one of the main problems related to phage application is the stability of bacterial viruses. Though many techniques have been used to sustain phage activity, novel tools are needed to allow long-term phage storage and application in versatile forms. In this study, we combined two well-known methods for bacteriophage immobilization. First, encapsulated phages were obtained by means of extrusion–ionic gelation, and then alginate microspheres were dried using the lyophilization process (freeze-drying). To overcome the risk of phage instability upon dehydration, the microspheres were prepared with the addition of 0.3 M mannitol. Bacteriophage-loaded microspheres were stored at room temperature for 30 days and subsequently exposed to simulated gastric fluid (SGF). The survival of encapsulated phages after drying was significantly higher in the presence of mannitol. The highest number of viable bacteriophages exceeding 4.8 log_10_ pfu/mL in SGF were recovered from encapsulated and freeze-dried microspheres, while phages in lyophilized lysate were completely inactivated. Although the method requires optimization, it may be a promising approach for the immobilization of bacteriophages in terms of practical application.

## 1. Introduction

Antimicrobial resistance of bacteria has in recent decades become a worsening global problem. In light of the limited efficacy of frontline antibiotics and the decreasing number of new antibacterial drugs introduced onto the market each year, innovative antibacterial strategies are urgently needed. In this context, the application of bacteriophages may be a promising approach.

The potential of bacteriophages in the treatment of bacterial infections was perceived over a century ago; however, in recent years, a real boost in the development of phage-based antibacterial strategies has been observed. Phage activity against multidrug-resistant (MDR), extensively drug-resistant (XDR), and pandrug-resistant (PDR) bacterial strains has been confirmed in many studies [1,2], and the potential of phage therapy in the “post-antibiotic era” has been noted [3,4,5,6].

Despite evidence of the efficiency of bacteriophages and the undisputed advantages of phage treatment over pharmacology-based antibacterial strategies, there are no specific regulatory agreements around phages at the moment [4]. Currently, no phage products are approved for human use by the European Medicines Agency (EMA) or the Food and Drug Administration (FDA) [7], and phage therapy is mostly practiced on a compassionate basis [8]. During the last two decades, significant progress in phage studies has been evident, but we still need to identify and fill gaps in the knowledge, complement experience, and optimize methods and practical tools to make this form of treatment accessible for patients. 

One of the limitations in the application of phage preparations is the stability of the viruses during passage through the gastrointestinal tract. Although phage preparations may be easily used in the forms of aerosols, compresses, and ear or eye drops, the oral administration of bacteriophages is associated with the increased risk of treatment inefficiency due to phage inactivation in the stomach environment [9]. This increased sensitivity of bacteriophages to adverse stomach conditions entails the selection of highly resistant phages or the administration of drugs neutralizing acidic pH during therapy [10,11]. Another aspect is phage stability during storage. Phages, as with other protein-based macromolecules, are prone to protein misfolding, aggregation, and denaturation [12]. Storage in liquid forms at 4 °C seems to be the most effective; however, it may not be convenient for commercial approaches. Moreover, considering the literature data and laboratory experience, survivability in these conditions may also be highly variable across bacteriophages [12].

Research in recent years has shown that using different techniques of immobilization may significantly improve phage stability in the stomach environment as well as during storage [13,14]. However, the golden mean is needed—a method which would provide broadly defined phage stability and at the same time a formulation which is convenient and patient-friendly. 

The objective of the study was to develop a method for obtaining a phage preparation in the form of a dry powder, making it possible to retain phage viability during storage and subsequently in an acidic stomach environment. We have combined two well-known techniques for phage immobilization. In the first stage, we encapsulated phages with an extrusion method. Then, to obtain the dry formulation, we used lyophilization (freeze-drying), a technique which is frequently used for the immobilization of molecules and microorganisms [14]. Since the dehydration of bacteriophage preparations may relate to significant titer loss, at the encapsulation stage, we used mannitol as an excipient, the protective properties of which we have demonstrated in previous studies [15]. Although the proposed method requires further optimization, we believe it may be a promising approach for the immobilization of bacteriophages in terms of practical application. 

## 2. Materials and Methods

### 2.1. Bacteriophage and Bacteria

Bacteriophage T4 (DSM-4505) and its *E. coli* host strain (DSM-613) were obtained from the German Collection of Microorganisms and Cell Cultures (Leibniz Institute DSMZ, Braunschweig, Germany). Bacterial cultures were grown and maintained on Luria-Bertani (LB) broth. Bacteriophages were amplified in host bacteria according to [16]. Briefly, 10 mL of LB broth was inoculated with a single colony of *E. coli* and incubated overnight at 37 °C (150 rpm). Then, 5 mL of phage lysate was added and the phage-bacteria culture was incubated for the subsequent 24 h in the same conditions. The culture was centrifuged (5000 rpm, t = 10 min, 20 °C) and the supernatant was filtered through a 0.22 µm syringe filter (Merck Millipore, Darmstadt, Germany). The filtrate was introduced into a 20-h host culture in 100 mL of liquid LB medium and incubated for 24 h at 37 °C with shaking (150 rpm). The culture was centrifuged (5000 rpm, t = 10 min, 20 °C) and filtered through a 0.22 µm syringe filter (Merck Millipore, Germany). Phage titer was determined by the double-agar-layer method (plaque assay) [17].

### 2.2. Encapsulation of Bacteriophage T4

Ca-alginate microspheres were prepared using an extrusion method adapted from [15] and modified. Bacteriophages were encapsulated in alginate (Sigma-Aldrich, Darmstadt, Germany) or alginate with mannitol (Sigma Aldrich, Germany) capsules using encapsulator B-390 (Büchi Labortechnik AG, Flawil, Switzerland). Some 30 mL of T4 lysate was mixed with 90 mL of 2% water solution of sodium alginate. Then, the mixture was extruded through the nozzle with a diameter of 150 µm, 300 µm or 450 µm into the 0.1 M CaCl_2_ or 0.1 M CaCl_2_ supplemented with 0.3 M mannitol at 23 °C. The following parameters of the encapsulation process were used: air pressure 150 mbar, frequency 800 Hz, and stream height 30 cm. The beads were kept in the solution for 20 min and were allowed to harden. Then, the formed beads were washed with distilled water. The shape and diameter of the capsules were investigated under the light microscope (Zeiss Axio Vert.A1 for wet microspheres and Leica DMI1 for dry microspheres).

### 2.3. Lyophilization of Encapsulated Bacteriophage T4

A total of 1 g of beads with bacteriophage T4 and 1 mL of T4 lysate were introduced into vials of 2 cm diameter, which were subsequently placed on the shelf in the freeze-dryer (FreeZone Triad Cascade Benchtop Freeze Dry System, Labcono, Kansas City, MO, USA) and pre-frozen to −36 °C at 0.5 °C/min. The process of freeze-drying was carried out at −36 °C, 0.20 mbar for 19 h, followed by a temperature shift to −10 °C and pressure of 0.18 mbar for 1.5 h, and finally +5 °C, 0.16 mbar for 4 h. The vials were stored at 23 °C for 30 days. 

### 2.4. Release of Phages from Microspheres

To verify the process of the release of phages from microspheres, they were suspended in microsphere-breaking solution (MBS), composed of 50 mM sodium citrate, 0.2 M sodium bicarbonate, and 50 mM Tris-Cl, pH 7.5 [18]. A total of 1 g of beads with bacteriophages were introduced into the bottles containing 99 mL of MBS, and incubated at 37 °C for 180 min with shaking (150 rpm). To assess the viability of the released phages, 1 mL of relevant samples were collected and phage titer was determined with the double-agar-layer method [17]. The efficiency of phage T4 encapsulation was calculated as the number of phage particles released from microspheres divided by the number of phages initially added to the solution and multiplied by 100.

### 2.5. Stability of Encapsulated Bacteriophages in the Simulated Gastrointestinal Conditions

For the encapsulated phage, resistance and survival in acidic conditions were evaluated using simulated gastrointestinal fluids according to [15]. Simulated gastric fluid (SGF) contained 2.05 g/L NaCl, 0.37 g/L KCl, 0.6 g/L KH_2_PO_4_, 0.11 g/L CaCl_2_, 0.05 g/L bile, 0.1 g/L lysozyme, and 3 g/L pepsin (Sigma-Aldrich). The pH of the solution was adjusted to 2.5. A total of 1 g of fresh microspheres (dried beads equivalent to 1 g of fresh beads) were introduced into 9 mL of SGF and incubated for 60 min at 37 °C with shaking at 200 rpm. After incubation, the beads were rinsed twice with distilled water. The samples already incubated in 9 mL of SGF were suspended in simulated intestinal fluid (SIF) (pH 7.5) composed of 25.2 g/L NaHCO_3_, 40 g/L bile, 3.5 g/L pancreatin, and 0.1 g/L trypsin (Sigma-Aldrich) and incubated for 180 min at 37 °C with shaking (200 rpm). Samples at the volume of 500 μL were collected and phage titer was determined with the double-agar-layer method at selected time intervals [17]. All solutions imitating the ingredients of the gastrointestinal environment were pre-warmed to 37 °C before use.

### 2.6. Statistical Analysis

All experiments were repeated three times independently. Data were expressed as means ± standard deviations (SD). Statistical analyses were performed using STATISTICA ver. 13 (TIBCO Software Inc., Palo Alto, CA, USA). Statistically significant differences were calculated with a one-way ANOVA and Tukey’s post-hoc test. The differences were considered significant at a *p*-value of <0.05.

## 3. Results

### 3.1. Encapsulation of Bacteriophage T4

**Figure 1:**
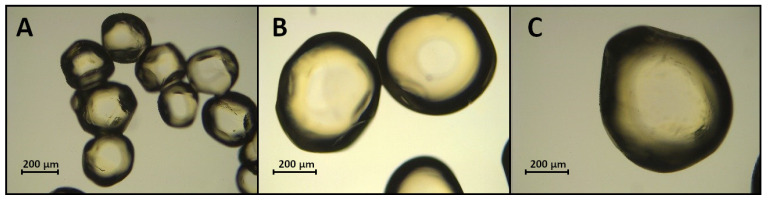
Optical micrograph of microspheres produced using (**A**) 150 µm, (**B**) 300 µm, (**C**) and 450 µm nozzle sizes.

The encapsulation process generated uniform microspheres with a smooth structure independent of the nozzle size used and the addition of 0.3 M mannitol (Figure 1). For a given viscosity of the encapsulated mixture and process parameters, the size of the beads produced generally exceeded twice the nozzle size (Table 1). The initial phage titer was adjusted to 9.63 log_10_ pfu/mL. Phages encapsulated in wet microspheres were found to fully maintain their viability and phage titers did not differ between both variants (Table 1). Encapsulation efficiency was measured in wet microspheres and showed effective immobilization of phage particles with a minimal value of 98.9% for bacteriophage T4 immobilized in microspheres obtained with a 450 µm nozzle (Table 2). 

### 3.2. Lyophilization of Bacteriophage T4

**Figure 2:**
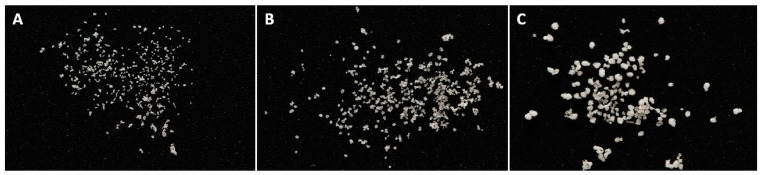
The image of freeze-dried microspheres produced using (**A**) 150 µm, (**B**) 300 µm, (**C**) and 450 µm nozzle sizes.

Lyophilized microspheres were of more irregular shapes and decreased diameters compared to wet beads (Figure 2). The mean diameter of the beads also corresponded to the nozzle used (Table 2). The largest particles achieved with a nozzle of 450 µm did not exceed a size of 0.53 mm.

Bacteriophages encapsulated without excipient were fully inactivated after the lyophilization process, while the addition of mannitol allowed for the retaining of phage viability exceeding 7.2 log_10_ pfu/mL. The lyophilization process induced a loss of at least 3 log_10_ pfu/mL of nonencapsulated phages (*p* > 0.05)(Table 3). 

### 3.3. Viability of Encapsulated Bacteriophage T4 after Storage

To determine the shelf-life of encapsulated phages, microspheres with phage T4 were stored for 30 days at 23 °C. Compared with the wet form, lyophilization decreased the recovery of stored encapsulated phages by about 3 log_10_ pfu/mL. However, a slight difference (*p* > 0.05) in phage titer over a 30-day storage time was observed between many of the tested variants of the dried alginate–mannitol microsphere. Phages remained viable in the lyophilized alginate–mannitol microsphere during storage, with the phage titer exceeding 6 log_10_ pfu/mL (Table 4). 

### 3.4. Stability of Immobilized Phage T4 in Simulated Gastrointestinal Conditions

The viability of encapsulated and lyophilized bacteriophages in SGF was determined right after the immobilization of the viruses and after 30 days. Wet beads without mannitol allowed for the retaining of the phage activity with a reduction of 1 log_10_ pfu/mL after incubation in SGF. We observed a release of >9 log_10_ pfu/mL bacteriophage particles from alginate–mannitol microspheres. Dry beads without mannitol were ineffective in protecting bacteriophages, and phages were completely inactivated after 30 min of incubation. Phages microencapsulated in dry spheres with mannitol were more resistant to gastric acids, and the titer of released phages was over 5 log_10_ pfu/mL and 4 log_10_ pfu/mL after 0.5 h and 1 h incubation in SGF, respectively.

Since the stability of encapsulated bacteriophages in wet variants had previously been confirmed, only the dry form of the preparation was subjected to further experiments. The T4 phage in dry microspheres was again exposed to SGF after 30 days of storage in undemanding conditions (23 °C). A significant reduction in phage titer by ~2 log_10_ units was observed compared to the first experiment in SGF (*p* > 0.05). However, a significant portion of viable phages exceeding 4.8 log_10_ pfu/mL were successfully released from 30-day alginate–mannitol microspheres (Table 5). At the same time, lyophilized bacteriophages were unstable over time, resulting in a complete loss of titer after incubation in SGF.

## 4. Discussion

One of the main limitations of the widespread use of phage therapy is the sensitivity of bacterial viruses to low pH, as orally administrated phages significantly decrease in activity in the stomach environment [19]. Therefore, the objective of this study was to prepare functional phage-based preparations, which are stable during storage and passage through the simulated conditions of the gastrointestinal tract. The application of different immobilization strategies in terms of phage stabilization has recently been extensively explored [14,20,21,22]. Here, we combined two commonly used techniques for immobilization of bacterial viruses, namely encapsulation and freeze-drying, to extend phage stability in time and subsequently in the unfavorable conditions of stomach acid.

Encapsulation ensures high immobilization efficiency and improves the physicochemical properties of the product (e.g., solubility, dispersibility, flowability). Equally importantly, encapsulation provides a controlled release of active components [23]. We demonstrated a marginal loss of phage particles resulting in high loading efficiency exceeding 9.4 log_10_ pfu/mL in microspheres produced using different nozzle sizes. Comparable efficiency was previously reported for alginate microspheres with the *E. coli*-specific phage UFV-AREG1 [24] and Felix O1 and K phages infecting *Salmonella* spp. and *S. aureus*, which were immobilized in whey–alginate microspheres [25,26]. A slightly lower efficiency of 93–95% was recorded in the first published tests for the production of alginate microspheres [18,27].

However, phages can undergo physical stresses in hydrated environments, which can lead to aggregation of the virions and a decrease in phage titer [28]. Moreover, the liquid formulation is not convenient for handling. Due to the intended use of the preparation, it is particularly desirable to maintain the stability of phages in capsules, bypassing the cold chain. Regardless of the method adopted, drying constitutes a key issue for phage survival. Freeze-drying makes it possible to obtain shelf-stable phage preparations, but this method alone does not protect phage viability in the adverse environment of the digestive system [29]. We have previously made a similar observation using less aggressive air-drying. However, the choice of excipient has proven to be essential for phage protection from drying and after incubation in gastrointestinal juice [15]. Here, encapsulated phages lyophilized with no excipient were fully inactivated, while the addition of mannitol allowed for the retaining of the phage titer, exceeding 7.0 log_10_ pfu/mL for all tested nozzles. Interestingly, lyophilized lysate retained a phage titer at a similar level, and it was stable for the 30 days of storage. This observation is consistent with other published reports suggesting that bacterial viruses may partially retain activity after lyophilization even with no cryoprotectants [30,31,32]. In contrast to these findings, some scientists observed nearly complete phage inactivation after this process when no excipient was applied [33,34]. Presumably, phages may vary among themselves with respect to sensitivity to the conditions of freeze-drying, which entails the optimization of the method [35]. To standardize the process, different excipients, e.g., sucrose and trehalose, are often added to phage suspensions to significantly improve the stability of the lysates [28,36,37,38,39]. In the present study, an excipient has been added at the stage of encapsulation, and the proposed scheme for preparing dried microspheres has no equivalent in the literature on phage encapsulation.

Bacteriophages encapsulated in alginate–mannitol microspheres and subsequently freeze-dried retained their activity above 7.2 log_10_ pfu/mL after storage. Data on the effects of polyhydric alcohols used to bind proteins are limited [40]. Mannitol’s protective properties may partially be the result of phage entrapment in the vitrified protectant matrix, which limits molecular mobility and maintains the structural stability of viruses [41]. However, according to some theories regarding spray-dried or freeze-dried bacteriophages, dried mannitol is dominated by a crystalline structure that may have a destabilizing effect on phage proteins and induce protein unfolding upon dehydration [42,43]. In fact, for the long-term stabilization of proteins, additives that come in an amorphous form are recommended. However, recrystallization of the dried phage preparation with a high titer reduction has been demonstrated when other excipients were used, e.g., trehalose, considered to represent an amorphous structure [44]. Despite the tendency of mannitol to form crystalline structures, its protective properties were significantly improved in both the presence of other polymers and by modifying the concentration [40]. Fully amorphous mannitol has been detected for the inhalable dry biotherapeutics of the calcitonin preserved an excipient content of 50% (*w*/*w*) [45]. In our study, a relatively low concentration of mannitol added to the encapsulation matrix most probably leads to the conformational stability of phage proteins despite the amorphous or crystalline phase particle form. This is reflected by the retained lytic activity of phages after lyophilization exceeding 7.2 log_10_ pfu/mL. Similar results have been presented in our previous work on phage encapsulation [15]. Then, mannitol is assigned a greater role as a bulking agent, which helps maintain the appropriate formulation and ensures mechanical strength. Moreover, mannitol does not exhibit hygroscopic properties, so phages enclosed in samples with low moisture content may maintain better stability during storage [46]. Data regarding the combined use of two immobilization techniques in the development of stable-over-time phage formulation are scarce. However, the results are consistent with the observations of Puapermpoonsiri et al. (2009), who used emulsification and lyophilization to obtain shelf-stable preparations of *Staphylococcus aureus* and *Pseudomonas aeruginosa* phages. Active phages were detected after release following 1, 3, and 7 days of storage [47].

The experimental model adopted in this study included the exposition of immobilized and stored phages to the environment of simulated gastric fluid. Although the titer of the phages released from the alginate-mannitol beads in SIF has significantly dropped compared to the initial concentration, active phages were still found at levels of 4.53 log_10_ pfu/mL, 4.84 pfu/mL and 5.34 log pfu/mL for microspheres of approximately 520 µm, 343 µm, and 220 µm. When comparing the survival of phages reported in the previous study [15], the air-drying of alginate beads resulted in comparative protective benefits for phages exposed to SIF. Up to 70% of phage titer remained active in the simulated gastric environment after storage. However, the air-drying technique cannot be directly compared to the freeze-drying of this study. Here, the significance of replacing the drying procedure lies in the fact that lyophilization is a widely adopted process in industrial drying, and allows for greater control of processing conditions. More importantly, as a well-understood dehydration strategy, lyophilization complements high-yield encapsulation, which in turn allows for precise size control. In the context of practical application, it has been suggested that small pellets less than 2 mm in size could be emptied from the stomach quite rapidly; thus, phages may not be greatly affected by the low pH of gastric acids [48]. Moreover, larger capsules may have a negative impact on the sensory properties of the product to which they are added [49]. For small animals such as mice and rats, small microparticles with comparable sizes below 0.5 mm have been suggested to be particularly useful for phage delivery by oral gavage during routine in vivo preclinical studies [50]. Validation of the size in relation to the target organism would be necessary to maintain optimal carrier protection. Despite the decrease in the number of viable phage particles, the obtained titer has been indicated by other scientists as an efficient therapeutic dose [51,52]. It is likely that using the preparations with initial higher phage titers would allow us to obtain higher titers after immobilization and incubation in SIF. It is worth noting that phages in the lyophilized lysate after storage and incubation in the acidic environment were completely inactivated. Furthermore, active phages were not detected in the case of alginate-only microspheres.

Despite numerous advancements in the development of phage stabilization methods, seeking procedures allowing us to obtain functional preparations still fits into the current trends of phage studies. The results of this study indicate that the subsequent application of encapsulation and lyophilization processes may be a promising approach in obtaining functional, shelf-stable phage preparation for oral application. Although the procedure requires optimization, it may be a good starting point for future studies. 

## 5. Conclusions

The growth of antibiotic resistance has become one of the most persistent public health problems. Currently applied methods for the elimination of microorganisms have become unreliable, and novel strategies that can replace conventional treatments are urgently required. Bacteriophage therapy is unquestionably considered one of the most promising alternatives to classical antibacterial approaches. However, despite the proven efficiency of phages and many other advantages of the application of bacterial viruses, this form of therapy still has some limitations. One of these limitations is phage instability while passing through the gastrointestinal tract after oral administration and during storage. 

In this study, we proposed a two-stage protocol based on encapsulation and subsequent lyophilization of bacteriophages, in order to develop a stable form of phage delivery intended for oral use. Encapsulation in alginate-based microspheres has previously been shown to be effective in protecting phages in the adverse environment of the stomach, while lyophilization is routinely used for the preparation of stable dry formulations of different microorganisms. We demonstrated that a combination of these methods results in the increased stability of phages during storage and then after incubation in a medium simulating the stomach environment. In this study, we also confirmed the protective properties of mannitol. The addition of mannitol at the stage of phage encapsulation turned out to be essential to achieving a high number of active phages in the formulation. Further investigation of excipient interactions during encapsulation and subsequent freeze-drying should be performed with respect to the physicochemical structure of the material used. Although the proposed method requires additional optimization, we consider it a promising starting point for future studies.

## Figures and Tables

**Table 1:** Activity of bacteriophage T4 after encapsulation in alginate and alginate–mannitol microspheres.

Nozzle Size [µm]	Diameter of Beads		Bacteriophage T4 Titer [log_10_ pfu/mL]			
	[µm]	SD	0.1 M CaCl_2_	SD	0.1 M CaCl_2_ 0.3 M Mannitol	SD
150	341	22	9.56 ^a^	±0.248	9.70 ^a^	±0.175
300	523	21	9.46 ^a^	±0.178	9.46 ^a^	±0.204
450	717	20	9.44 ^a^	±0.175	9.44 ^a^	±0.227
			Initial phage titer 9.63 log_10_ pfu/mL ^a^			

Means with the same superscript letter show no significant difference at *p* < 0.05.

**Table 2:** Encapsulation efficiency of bacteriophage T4.

Nozzle Size [µm]	Encapsulation Efficiency [%]			
	0.1 M CaCl_2_	SD	0.1 M CaCl_2_ 0.3 M Mannitol	SD
150	99.4 ^a^	±1.538	100 ^a^	±1.937
300	100 ^a^	±1.482	99.8 ^a^	±1.724
450	98.9 ^a^	±1.724	99.1 ^a^	±0.970

Means with the same superscript letter show no significant difference at *p* < 0.05.

**Table 3:** The activity of encapsulated bacteriophage T4 after the lyophilization process.

Nozzle Size [µm]	Diameter of Beads		Bacteriophage T4 Titer [log_10_ pfu/mL]		
	[µm]	SD	0.1 M CaCl_2_	0.1 M CaCl_2_ 0.3 M Mannitol	SD
150	224	17	0	7.52 ^a^	±0.004
300	343	15	0	7.50 ^a^	±0.082
450	524	24	0	7.24 ^a^	±0.076
			Lyophilized phage lysate: 6.24 log_10_ pfu/mL ^b^		
			Initial phage titer: 9.63 log_10_ pfu/mL ^c^		

Means with the same superscript letter show no significant difference at *p* < 0.05.

**Table 4:** Activity of immobilized bacteriophage T4 after 30 days of storage.

Nozzle Size	Bacteriophage T4 Titer [log_10_ pfu/mL]					
	Wet Microspheres CaCl_2_		Wet Microspheres CaCl_2_ and Mannitol		Dry Microspheres CaCl_2_ and Mannitol	
150	9.59 ^a^	±0.088	9.62 ^a^	±0.074	6.92 ^c^	±0.103
300	9.75 ^a,b^	±0.195	9.89 ^a^	±0.073	6.68 ^c,d^	±0.247
450	9.83 ^b^	±0.038	9.57 ^a^	±0.081	6.23 ^d^	±0.206
Lyophilized phage lysate: 6.13 log_10_ pfu/mL ^d^						
Initial phage titer: 9.63 log_10_ pfu/mL ^a^						

Means with the same superscript letter show no significant difference at *p* < 0.05.

**Table 5:** Activity of bacteriophage T4 after storage and incubation in SGF.

	Time of Incubation in SGF [h]	Nozzle Size	Bacteriophage Titer [log_10_ pfu/mL]		
			1 Day after Immobilization		30 Days after Immobilization
			Wet Microspheres	Dry Microspheres	Dry Microspheres
0.1 M CaCl_2_	1	150	8.60 ± 0.167 ^b^	0	n.i.
		300	8.64 ± 0.191 ^b^	0	n.i.
		450	8.71 ± 0.100 ^b^	0	n.i.
0.1 M CaCl_2_ 0.3 M mannitol	0.5	150	9.63 ± 0.135 ^c^	5.28 ± 0.118 ^e^	
	1			5.68 ± 0.137 ^d^	5.34 ± 0.086 ^e^
	0.5	300	9.75 ± 0.180 ^c^	5.28 ± 0.153 ^e^	
	1			4.86 ± 0.125 ^f^	4.84 ± 0.241 ^f^
	0.5	450	9.55 ± 0.150 ^c^	5.27 ± 0.194 ^e^	
	1			4.82 ± 0.476 ^f^	4.53 ± 0.152 ^g^
Initial phage titer: 9.63 log_10_ pfu/mL ^a^					

Means with the same superscript letter show no significant difference at *p* < 0.05.

## Data Availability

All data generated or analyzed during this study are included in the published article.
